# Controlled Synthesis and Characterization of Micrometric Single Crystalline Magnetite With Superparamagnetic Behavior and Cytocompatibility/Cytotoxicity Assessments

**DOI:** 10.3389/fphar.2020.00410

**Published:** 2020-04-03

**Authors:** Claudia Geanina Farcas, Ioana Macasoi, Iulia Pinzaru, Marius Chirita, Marius Constantin Chirita Mihaila, Cristina Dehelean, Stefana Avram, Felicia Loghin, Liviu Mocanu, Virgil Rotaru, Adrian Ieta, Aurel Ercuta, Dorina Coricovac

**Affiliations:** ^1^Department of Toxicology, Faculty of Pharmacy, “Iuliu Hatieganu” University of Medicine and Pharmacy, Cluj-Napoca, Romania; ^2^Faculty of Pharmacy, “Victor Babes” University of Medicine and Pharmacy, Timisoara, Romania; ^3^Department of Condensed Matter, National Institute for Research and Development in Electrochemistry and Condensed Matter, Timisoara, Romania; ^4^Max F. Prutz Laboratories, Department of Structural and Computational Biology, University of Vienna, Vienna, Austria; ^5^Quantum Optics, Quantum Nanophysics and Quantum Information, Faculty of Physics, University of Vienna, Vienna, Austria; ^6^Faculty of Medicine, “Victor Babes” University of Medicine and Pharmacy, Timisoara, Romania; ^7^Electrical and Computer Science Department SUNY Oswego, Oswego, NY, United States; ^8^Faculty of Physics, West University of Timisoara, Timisoara, Romania

**Keywords:** single-crystalline, superparamagnetic, micrometric, healthy/tumor cells, viability

## Abstract

A new class of magnetite (Fe_3_O_4_) particles, coined as “Single Crystalline Micrometric Iron Oxide Particles” (SCMIOPs), were obtained by hydrothermal synthesis. Both the single Fe_3_O_4_ phase content and the particle sizes range, from 1 µm to 30 µm, can be controlled by synthesis. The notable finding states that these particles exhibit vanishing remanent magnetization (σr=0.28 emu/g) and coercive force (Hc=1.5 Oe), which indicate a superparamagnetic-like behavior (unexpected at micrometric particles size), and remarkably high saturation magnetization (σs=95.5 emu/g), what ensures strong magnetic response, and the lack of agglomeration after the magnetic field removal. These qualities make such particles candidates for biomedical applications, to be used instead of magnetic nanoparticles which inevitably involve some drawbacks like aglommeration and insufficient magnetic response. In this sense, cytocompatibility/cytotoxicity tests were performed on human cells, and the results have clearly indicated that SCMIOPs are cytocompatible for healthy cell lines HaCaT (human keratinocytes) and HEMa (primary epidermal melanocytes) and cytotoxic for neoplastic cell lines A375 (human melanoma) and B164A5 (murine melanoma) in a dose-dependent manner.

## Introduction

Iron oxides manufactured as nanoparticles or microparticles are considered materials with multi-purpose biomedical potential that proved great results in different biomedical applications, as: drug-delivery carriers, cancer therapy (targeted therapy by applying an external magnetic field), hyperthermia, diagnostic agents (nuclear magnetic resonance – NMR, magnetic resonance imaging - MRI), tools for *in vitro* techniques (diagnostic separation, magnetorelaxometry), etc (Catalano, 2017). Application of iron oxides (of nanometric or micrometric size) in biomedical fields has considerably developed in the recent years, as well as human exposure, and it became mandatory for the novel synthetized magnetic particles to be quantitatively analyzed from physico-chemically and toxicological perspectives.

The large variety of existing iron oxide particles (IOPs) on the market can be classified into ultra-small (USIOPs; 20 nm–50 nm diameters), small (SIOPs; 60 nm to cca. 250 nm), and micrometric (MIOPs; 0.9 μm and larger) synthesized by clustering superparamagnetic nanoparticles (Runge, 1996; Carroll et al., 2010). There is solid experimental evidence that nanoparticles smaller than 10 nm exhibit both toxicity risks, and the occurrence of physiological barriers for an enhanced permeability and retention (EPR) effect; these particles strongly interact with the immune system, and penetrate into capillaries (Hughes, 2015; Lauterwasser, 2015). The multiple challenges regarding nanoparticles biocompatibility, toxicological and immunological issues (Arias et al., 2018) determined the researchers to channel their interest in obtaining microparticles that possess similar features as nanoparticles (like superparamagnetism), but with an enhanced biocompatibility and low/absence of toxicity. Two options are mentioned in the literature: embedding thousands of individual SPIONs into micro-clusters or increasing every single particle dimension (Xie and Zhang, 2011). Mankia et al. (2011) verified the toxicological profile of MIOPs by performing animal studies and the results showed that neither tissue infarction, thrombosis, or vessel plugging *in vivo*, nor other noxious effects were noticed (Mankia et al., 2011). Moreover, it was demonstrated that liver and spleen cleared far more rapidly MIOPs from the blood circulation than USIOPs. Due to their size and incompressible nature, MIOPs are less susceptible to non-specific vascular egress or uptake by endothelial cells. By applying different methods of synthesis were obtained iron oxide microparticles with enhanced biological properties: agglomerations of magnetite nanoparticles with a superparamagnetic core (11.8 µm) and amoxicillin cover for the treatment of the spiral form of gram-negative bacteria *Helicobacter pylori* (Silva et al., 2009); MIOPs in the range of 1 µm—as contrast agents in mouse brain inflammatory pathology which enabled *in vivo* detection of the disease; larger MIOPs for cellular MRI imaging (Wu et al., 2006), characterization of vascular inflammatory disease (McAteer et al., 2008; Ye et al., 2008), molecular imaging of thrombosis (Von Zur Muhlen et al., 2009), molecular imaging of tissue ischemia (Akhtar et al., 2010) and as contrast agents for the detection of endovascular molecular targets by MRI (McAteer et al., 2011); magnetic oxide particle suspension in distilled water (10.82 μm average size) as MRI contrast agents (Mathieu and Martel, 2006). Nevertheless, it was reported that for molecular magnetic resonance imaging (mMRI), microparticles of iron oxide (MIOPs) create potent hypo intense contrast effects, especially due to their physical size (Mankia et al., 2011).

Despite the many advantages presented above, the major drawback in using micro-clusters resulted from multiple individual SPIONs is the small value for magnetic saturation (Ms) which means a weak magnetic response and all the disadvantages that arise due to a low response in MRI or the difficulty of handling them *via* an external magnetic field. In this context, our research group has focused on developing a controlled hydrothermal synthesis technique for producing “Single Crystalline Micrometric Iron Oxide Particles” SCMIOPs (from 1 µm to 30 µm), qualified for biomedical applications and able to overcome the above-mentioned limitations in using nanoparticles and micro-clusters. This report also presents an area of novelty regarding the cytocompatibility/cytotoxicity of SCMIOPs on both normal—keratinocytes and melanocytes and tumoral—human and murine melanoma cells by using specific *in vitro* methods such as viability assay and fluorescence staining.

## Materials and Methods

### Materials

#### Chemicals and Reagents

Analytical pure ferric ammonium sulphate FeNH_4_(SO_4_)_2_•12H_2_O (FAS), tetrasodium ethylenediaminetetraacetate (Na_4_EDTA), and urea (NH_2_)_2_CO were supplied by Fluka (Sigma-Aldrich) and used for Fe_3_O_4_ synthesis.

#### *In Vitro* Experiments

Dulbecco’s Modified Eagle’s Medium (DMEM) high glucose, fetal calf serum (FCS), saline phosphate-buffered (PBS), penicillin/streptomycin mixture, trypsin-EDTA solution, Trypan blue, Dermal Cell Basal Medium and Adult Melanocyte Growth Kit were purchased from Sigma Aldrich (Germany), Thermo Fisher Scientific (USA), and ATCC (American Type Culture Collection). The provider of MTT Cell Proliferation Assay Kit was Roche Applied Science (Mannheim, Germany).

### Methods

#### Synthesis and Characterization of SCMIOPs

Presently, SCIMIOPs were synthesized using a two-step method. Step one consists in the obtaining of Fe-EDTA complex using an aqueous solution of 1.05x10^-1^M FAS, 1.05x10^-1^M Na_4_EDTA, and 9.71x10^-1^ M urea. The Fe(III)EDTA complex formation is marked by the color change from purple to dark red. Step two consists in hydrothermal decomposition of Fe-EDTA. The solution was transferred into a 70 ml volume Teflon-lined stainless-steel autoclave and heated up to 230°C by a rate of 1.7°C/min. Three degrees of filling were selected for autoclaves: 50%, 60%, and 70%. For each filling level the high-pressure treatment time was progressively increased from 4 h to 40 h with a 2-hour growth rate. Abrupt cooling with cold water ensured the freezing of phase transitions inside the autoclaves. All the pH measurements indicated a value between 9.4 and 9.5 for the final solutions. The obtained microparticles were washed with bi-distilled water and dried two hours at 60°C.

The morphological examination of Fe_3_O_4_ crystals was carried out by scanning electron microscopy (SEM) using the Quanta 3D 200i, FEI Co. The chemical components were then identified by energy-dispersive X-ray spectroscopy (EDX) analysis. Structure analysis was performed at room temperature, using the X’Pert PRO MPD diffractometer (PANalytical) using Cu-Kα radiation (0.15418 nm, Ni filter) in θ:θ configuration. An AC hysteresigraph (Ercuta, 2020) was used to test the magnetic properties of the particles.

#### Cell Culture

The *in vitro* cytocompatibility/cytotoxicity tests were developed on two types of healthy cell lines and on two tumor cell lines: immortalized human keratinocytes (HaCaT - 300493; CLS Cell Lines Service GmbH), primary epidermal melanocytes (HEMa - ATCC^®^ PCS-200-013™), human melanoma (A375 - ATCC^®^ CRL-1619™), and murine melanoma (B164A5 – 94042254; ECACC). HaCaT, A375, and B164A5 cells were cultured in specific culture medium - Dulbecco’s modified Eagle Medium high glucose supplemented with 10% fetal calf serum and 1% penicillin/streptomycin solution. HEMa cells culture required Dermal Cell Basal Medium supplemented with Adult Melanocyte growth kit, 1% penicillin/streptomycin mixture, and 1% FCS. Throughout the experiments, the cells were maintained in a humidified incubator in standard conditions (5% CO_2_ at 37°C) and were passaged every other day. The cells were counted using Countess™ II Automated Cell Counter in the presence of Trypan blue.

#### Cell Viability Assessment

The cytotoxicity evaluation of Fe_3_O_4_ micrometric particles was performed according to the ISO standard 10993-5:2009 on Biological Evaluation of Medical Devices (https://www.iso.org/obp/ui/#iso:std:iso:10993:-5:ed-3:v1:en). The cells (1x10^4^ cells/200 µl culture medium) were seeded in 96-well culture plates with flat-bottom and allowed to attach until the confluence was appropriate (generally, for 24 h). The old medium was replaced by 100 µl fresh medium that contained different concentrations of Fe_3_O_4_ microparticles (25, 50, 100, 150, 250, 500, and 1000 µg/ml) and incubated for 24 h. The viability was assessed using MTT (3-(4,5-Dimethylthiazol-2-yl)-2,5-diphenyltetrazolium bromide) assay. In brief, a volume of 10 μl MTT reagent was added to each well and the plate was incubated for three hours at 37°C, followed by addition of 100 μl of solubilization buffer/well and incubation for 30 min at room temperature and dark. Further, the samples were spectrophotometrically analyzed at 570 nm, using a microplate reader (xMark™Microplate, Biorad). The results are presented as the mean % of viable cells compared to the control ± SD (n=3 for each concentration). The unstimulated cells were considered as control. The changes in cells morphology were monitored by Olympus IX73 inverted microscope under bright light illumination.

#### Prussian Blue Staining

This technique was performed in order to detect the localization of the SCMIOPs in cells monolayer. A number of 2x10^5^ cells/well were plated in 12-well culture plates to achieve a confluent culture cell monolayer. When the confluence was above 80%, the cells were stimulated with different concentrations of SCMIOPs (25, 50, 100, and 150 µg/ml) for 24 h. Thereafter, cells were washed two times with PBS, fixed with 4% paraformaldehyde at 4°C for 30 min, followed by staining at room temperature for 20 min with an equal volume of a freshly prepared mixture of 5% HCl in PBS and 5% potassium ferrocyanide. Cells were further counterstained with 1% neutral red solution for 5 min and destained with PBS (Jadhav et al., 2013). Cells were observed and pictured under bright field (BF) microscopy, at 40x magnification.

#### DAPI (4’,6-Diamidino-2- Phenylindole) Staining

In order to visualize the nuclear alterations specific for apoptosis induction, all the cell lines were stained with DAPI and analyzed under an inverted fluorescence microscope (Olympus IX73, Tokyo, Japan). A number of 1x10^6^ cells/well were seeded onto 6-well plates and were allowed to attach to the bottom of the well, overnight. The following day the old medium was removed, and the cells were treated for 24 h with a fresh medium containing different concentrations of SCMIOPs (25, 50, 100, and 150 µg/ml). Upon completion of the incubation period, the cells were washed twice with ice-cold PBS, fixed with 4% paraformaldehyde in PBS and permeabilized with 2% Triton-X/PBS for 30 min. The protocol was continued by a blocking step (30% FCS in 0.01% Triton-X), a washing step with PBS, and staining process with DAPI (300 nM) in a dark chamber. The cells were analyzed under a fluorescence microscope, at 40x magnification.

#### Statistical Analysis

Graph Pad Prism 6 and cellSens Dimensions v.1.8. software were used for the presentation and interpretation of the results. The results were expressed as the mean ± standard deviation (SD). One-way ANOVA analyze was applied to determine the statistical differences followed by Tukey post-test (* p <0.05; ** p < 0.01; *** p < 0.001).

## Results

### Characterization of SCMIOPs

**Figure 1:**
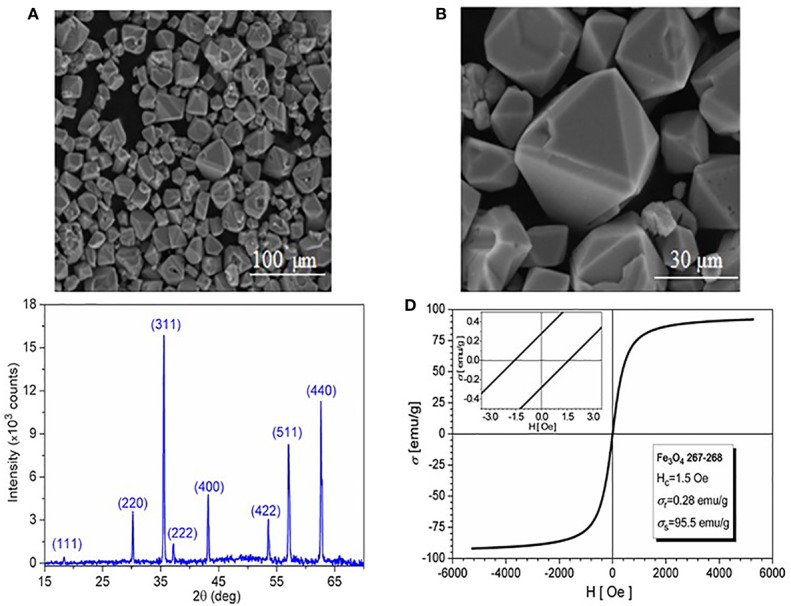
**(A)** SEM image of magnetite; **(B)** SEM image detail; **(C)** XRD spectrum; and **(D)** Hysteresis loop at room temperature.

All samples resulted during synthesis process were characterized. The samples obtained at 30 h of high-pressure treatment time in autoclave with a filling level of 50% were chosen for presentation, due to the lack of FeCO_3_ traces on the surface of Fe_3_O_4_ microcrystals. The SEM images of the 30 h magnetite microcrystals obtained from hydrothermal reaction are presented in **Figures 1A, B**. The microcrystals have a compact structure (no porosity observed), size between 1 µm and 30 µm, and morphologies that represent a combination of octahedral and dodecahedral faces. The XRD spectrum presented in **Figure 1C** allows the identification of the Fe_3_O_4_ after 30 h of high pressure-temperature treatment; the diffraction peaks were indexed using the ICSD (Inorganic Crystal Structure Database) reference code: 01-088-0315 for Fe_3_O_4_. The high purity of the 30 h final product is confirmed by the EDAX spectrum (chart not presented here), with no traces of Na, S, C, and N (which could result from EDTA and FAS decomposition).

The magnetic behavior at 300°K (the hysteresis loop) of the 30 h magnetite microcrystals is shown in **Figure 1D**. Fitting the hysteresis loop branches to Langevin-type functions, the saturation magnetization of the sample was estimated within ±3 emu/g accuracy to 95.5 emu/g. The vanishing values of both coercivity (Hc=1.5 Oe) and remanence (σr=0.28 emu/g) indicate superparamagnetic behavior.

### *In Vitro* Impact of SCMIOPs

**Figure 2:**
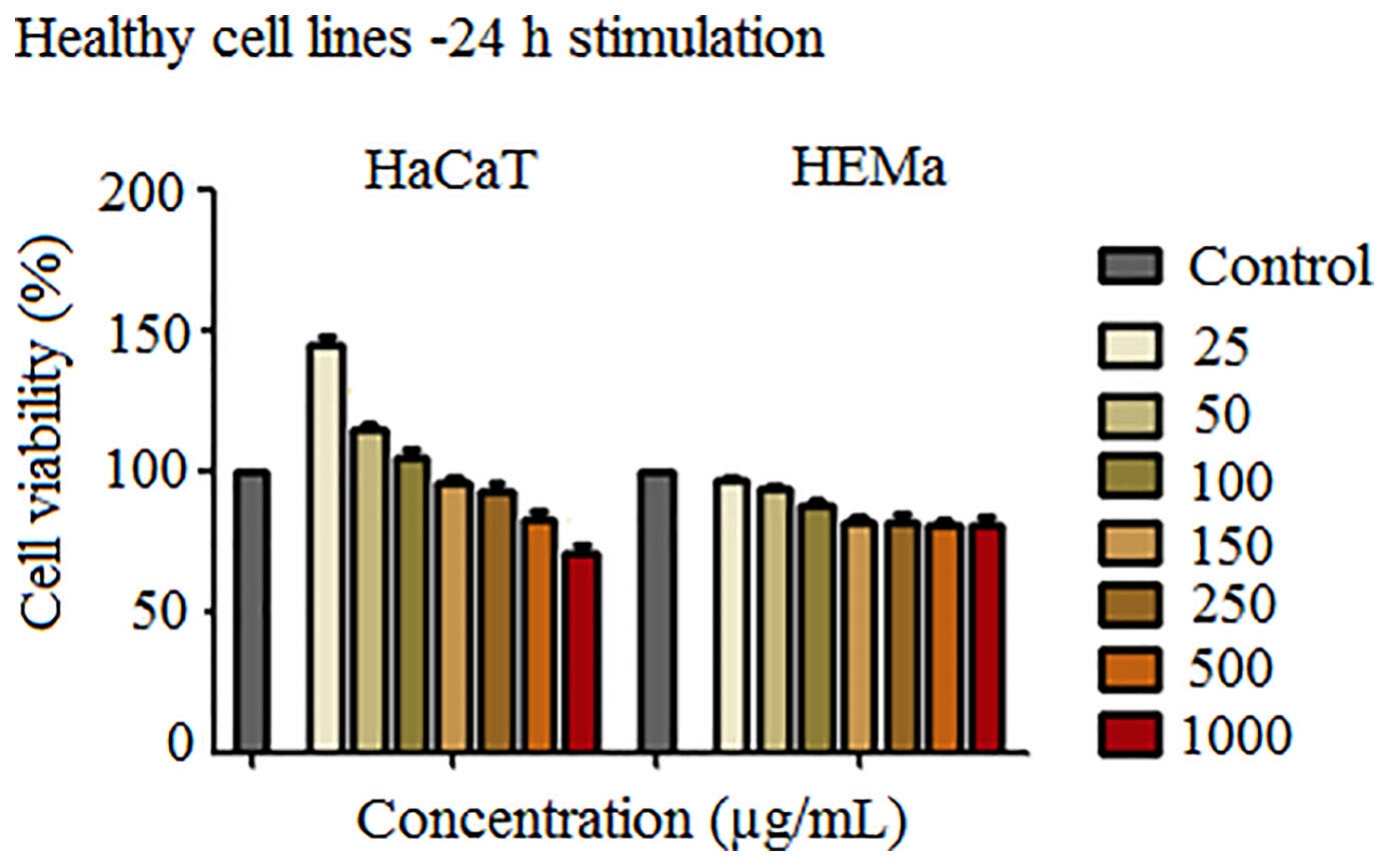
Cell viability assessment of SCMIOPs (25, 50, 100, 150, 250, 500, and 1000 μg/ml) on HaCaT and HEMa cells at 24 h post-stimulation by the means of MTT assay. The results are expressed as cell viability percentage (%) normalized to control cells (no stimulation). The data represent the mean values ± SD of three independent experiments performed in triplicate. One-way ANOVA analysis was applied to determine the statistical differences followed by Tukey post-test (**p < 0.01; ***p < 0.001).

Since the magnetite microparticles designed and synthesized in the present study are intended for biomedical applications, it is mandatory to verify their effects by performing *in vitro* assays to confirm or infirm their toxicological profile. The impact of SCMIOPs was tested for cytocompatibility on two healthy cell lines: HaCaT (immortalized human keratinocytes) and HEMa (primary epidermal melanocytes). In the case of HaCaT cells (**Figure 2** **- left panel**), the lower concentrations of SCMIOPs (25, 50, and 100 µg/ml) induced an increased cell viability, whereas higher concentrations (250, 500, and 1000 µg/ml) were associated with a decrease in the cell viability percentage in a concentration-dependent manner. The displayed viability rates were 92.18%, 82.52%, and 70.62%, respectively. At the 150 μg/ml, the viability has a value very close to the control sample.

HEMa cells proved to be more sensitive to SCMIOPs treatment (**Figure 2** **- right panel**) as compared to HaCaT cells; a decrease in the cell viability percentage starting with the lowest concentration tested (25 μg/ml) was observed. However, a significant reduction was recorded at 150 μg/ml (about 82%) when compared to control cells. Nevertheless, stimulation at higher SCMIOPs concentrations (250, 500, and 1000 μg/ml) led to a constant viability rate above 80%, similar to the one induced at 150 μg/ml, effect that is in contrast to the one observed in HaCaT cells which expressed a dose-dependent cell viability decrease.

**Figure 3:**
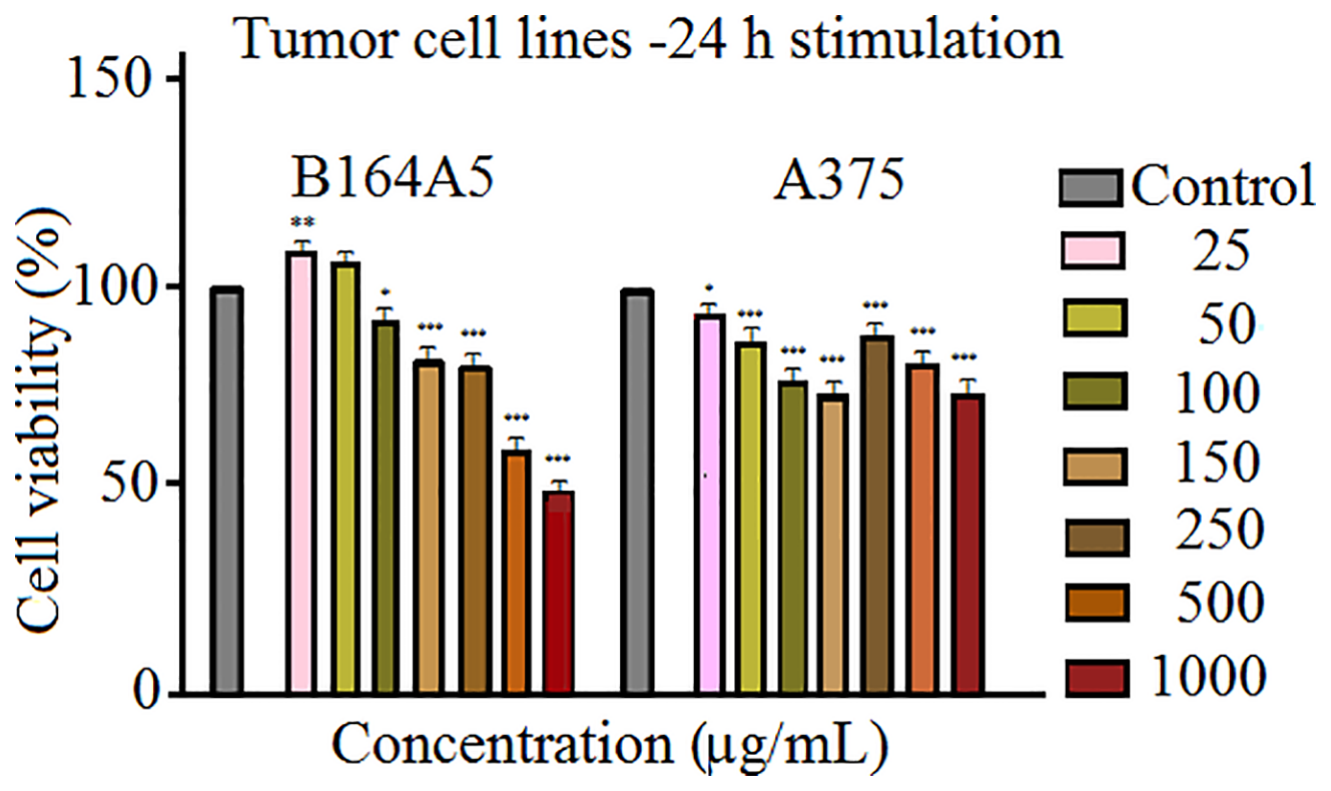
Cell viability assessment of SCMIOPs (25, 50, 100, 150, 250, 500, and 1,000 μg/ml) on B164A5 and A375 cells at 24 h post-stimulation by the means of MTT assay. The results are expressed as cell viability percentage (%) normalized to control cells (no stimulation). The data represent the mean values ± SD of three independent experiments performed in triplicate. One-way ANOVA analysis was applied to determine the statistical differences followed by Tukey post-test (*p < 0.05; **p < 0.01; ***p < 0.001).

The results obtained when the human - A375 and murine - B164A5 melanoma cells were stimulated with Fe_3_O_4_ microparticles (**Figure 3**) appear to be of particular interest. The magnetite microparticles induced a significant decrease of melanoma cells viability. The effect is more significant in the case of murine melanoma cells - B164A5 (**Figure 3** **- left panel**). A significant drop of cell viability starts at 100 μg/ml (about 92%) and decreases in a concentration-dependent manner to 49.87% (at 1000 μg/ml). The effect on human - A375 melanoma cell line (**Figure 3** **- right panel**) did not display a linear dependence with Fe_3_O_4_ concentration. However, A375 cells seemed sensitive to Fe_3_O_4_ treatment, even at the lowest concentration tested (25 μg/ml), showing a cell viability about 92%.

However, the viability rate decreased to 73% after treatment with 150 μg/ml SCMIOPs. Above this concentration an increase of cell viability was observed after stimulation at 250 and 500 μg/ml (89.26% and 80.21% viable cells, respectively). Nevertheless, the highest concentration (1000 μg/ml) induced the most significant decrease in A375 cell viability (about 71%).

### Cell Morphology and Apoptotic Markers

The morphological aspect of the cells was monitored by bright field (BF) microscopy at 0, 3, 6, and 24 h post stimulation. The most significant morphological changes were recorded at 24 h post-stimulation. Accordingly, assessment of possible nuclei alterations *via* DAPI staining was performed at the same time (24 h post-stimulation).

**Figure 4:**
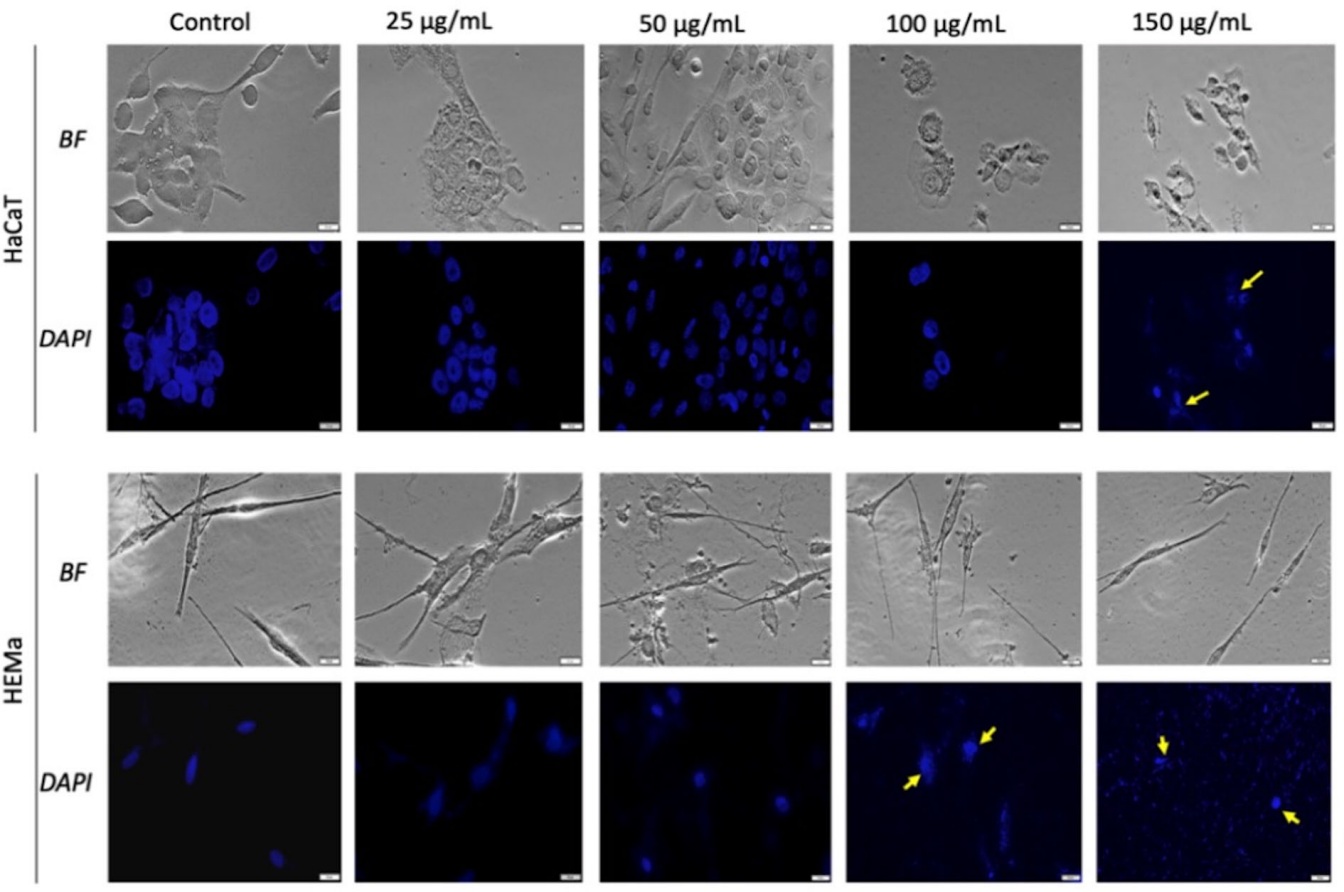
Healthy - HaCaT and HEMa cells treated with SCMIOPs at different concentrations (25, 50, 100, and 150 µg/ml) for 24 h. Bright field (BF) microscopy was employed to analyze morphological changes; DAPI staining (blue) was performed to evaluate cell nuclei modifications. Scale bars represent 20 µm.

**Figure 5:**
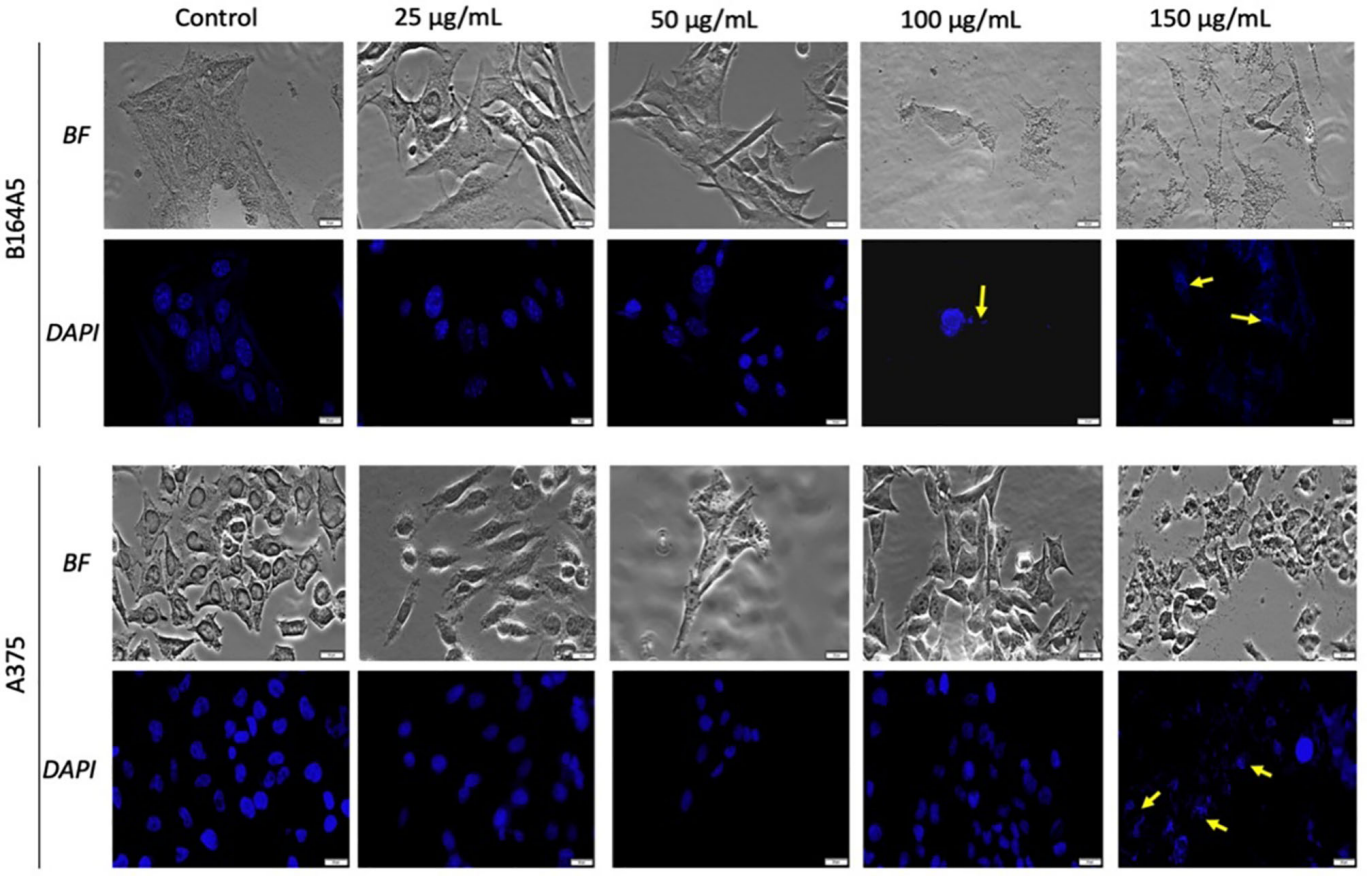
Melanoma - B164A5 and A375 cells treated with SCMIOPs at different concentrations (25, 50, 100, and 150 µg/ml) for 24 h. Bright field (BF) microscopy was employed to analyze morphological changes; DAPI staining (blue) was performed to evaluate cell nuclei modifications. Scale bars represent 20 µm.

As shown in **Figures 4** and **5**, the control cells display well defined elongated shape for HaCaT, HEMa, and B164A5 cell lines and a round shape for human melanoma A375 cells; also, the cells have a large nucleus with uniform chromatin density. Modification of cell morphology with apoptotic characteristics (cell shrinkage, DNA fragmentation and chromatin condensation) was observed in all cell lines, after SCMIOPs stimulation with 150 µg/ml. Nevertheless, HEMa and B164A5 cells were also affected by a lower concentration (100 µg/ml). Apoptotic aspects are marked with yellow arrows (**Figures 4** and **5**).

### SCMIOPs Detection Within the Cell Monolayer

**Figure 6:**
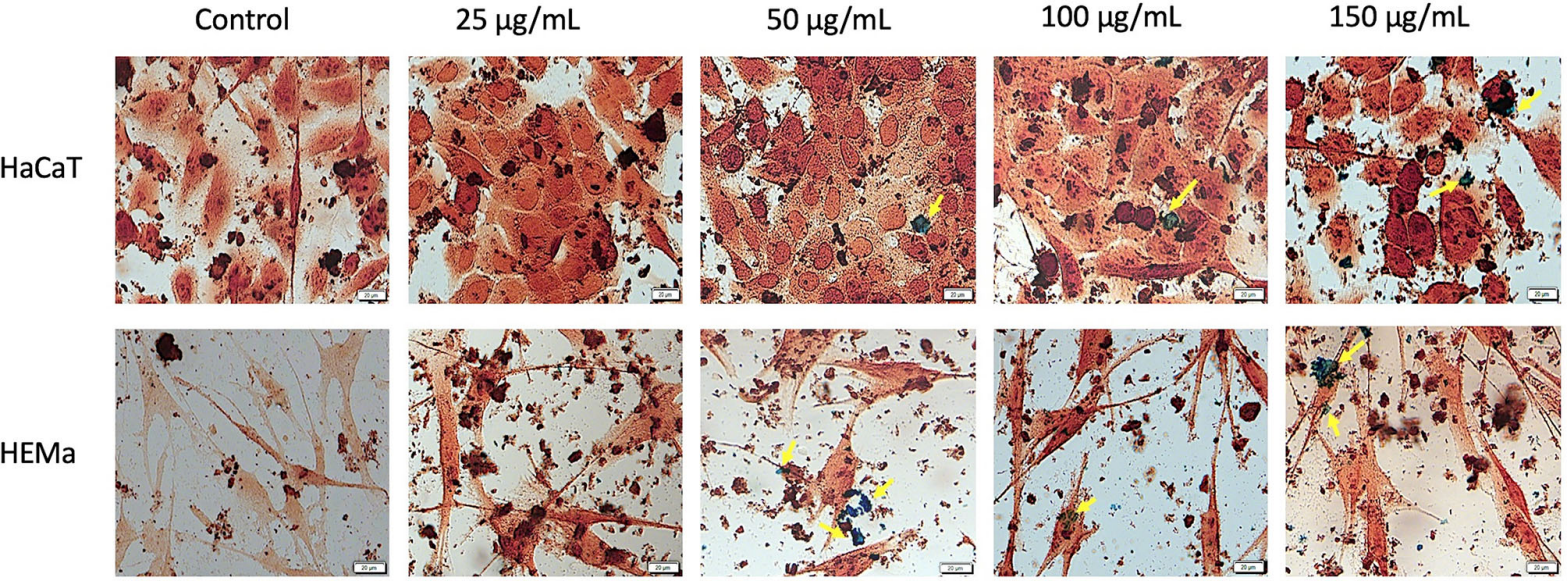
Healthy - HaCaT and HEMa cells incubated with different concentrations (25, 50, 100, and 150 µg/ml) of SCMIOPs for 24 h and stained with Prussian blue reagent. Pictures were taken using Bright field (BF) microscopy. Scale bars represent 20 µm.

**Figure 7:**
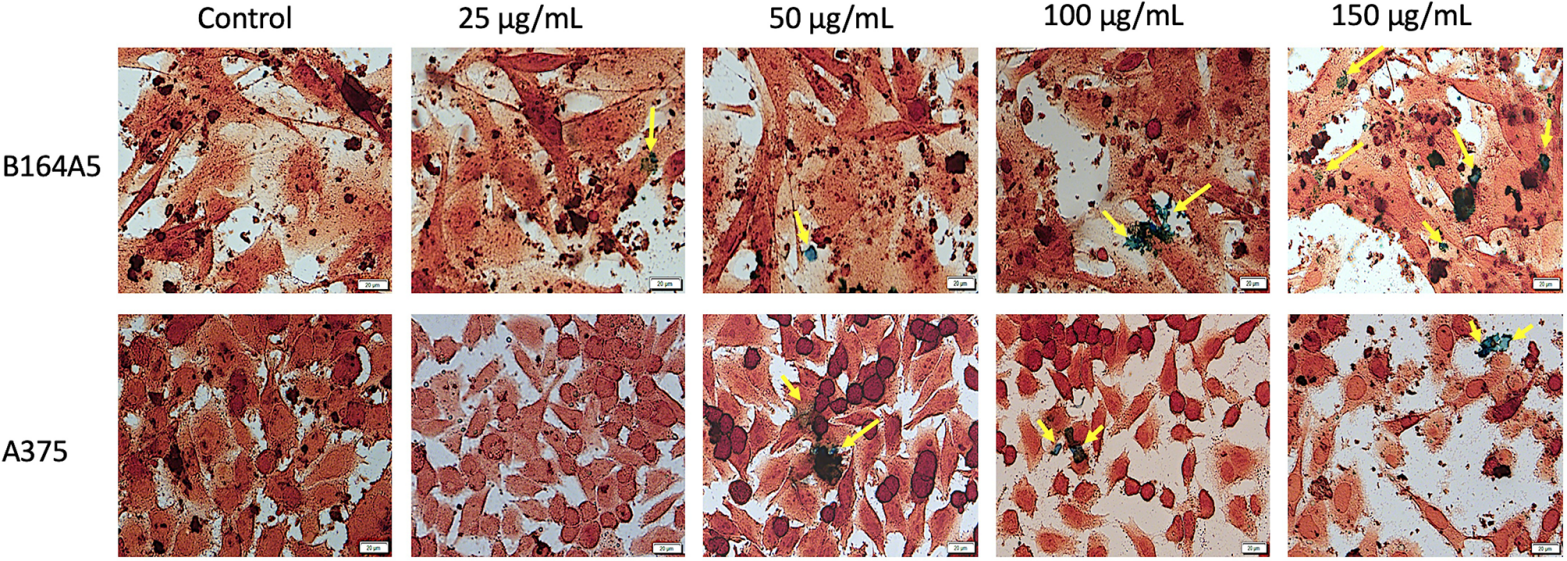
Tumor - B164A5 and A375 cells incubated with different concentrations (25, 50, 100, and 150 µg/ml) of SCMIOPs for 24 h and stained with Prussian blue reagent. Pictures were taken using Bright field (BF) microscopy. Scale bars represent 20 µm.

Prussian blue assay is a specific technique used to determine the *in vitro* cellular iron uptake, highlighting the presence of SCMIOPs by staining them blue. However, a large number of Fe_3_O_4_ microparticles did not penetrate the cell membrane (see yellow arrows in **Figures 6** and **7**). This happens mainly when HaCaT and A375 cells are present in the culture medium, whereas in the case of HEMa and B164A5 cells, the microparticles seem to be adherent to cells membranes.

### SCMIOPs Stability in Cell Growth Medium

Agglomerations of Fe_3_O_4_ microparticles were macroscopically observed during the *in vitro* experiments. Therefore, the stability of SCMIOPs’ was examined in complete cell medium (Dulbecco’s modified Eagle Medium supplemented with 10% fetal calf serum). The wells containing the cell medium with SCMIOPs at 25, 50, 100, and 150 μg/ml were pictured initially and at 24 h post incubation (figures not shown here). The plate was maintained in the same conditions as the ones used for *in vitro* experiments. It was observed that the Fe_3_O_4_ microparticles agglomerated after 24 h. The fact may be caused by the high salt concentration in the medium which can induce attractive electrostatic forces between microparticles leading to aggregates formation (Park et al., 2014).

## Discussions

Magnetite is known as the only metallic compound produced by the human body (a few hundred micrograms) and other living organisms as biochemical precipitate that possesses electrical conductivity leading to strong interactions with external magnetic field. These interactions determine different cellular effects, and thus far it is not clearly defined the impact of magnetite particles on human health (Kirschvink et al., 1992; Giere, 2016). Each type of magnetic particle has both advantages and disadvantages, especially depending on their properties which play a key role in biomedical field. The main factors which significantly influence these properties are related to the methods of synthesis and the [Fe2+]/[Fe3+] ratio. The hydrothermal process can be employed to control the size and the corresponding structural and magnetic properties which leads to an increased saturation magnetization as nanoparticles increase in size and also, to the formation of high crystalline iron oxide nanoparticles with increasing reaction time and temperature (Ozel and Kockar, 2015; Ozel et al., 2015). On the other hand, an optimization of magnetic saturation is related likewise for nanoparticles produced by co-precipitation in air atmosphere by using orthogonal design technique while particle sizes and magnetization increased with the increase of [Fe2+]/[Fe3+] ratio (Karaagac and Kockar, 2012; Karaagac and Kockar, 2016). Since iron microparticles present a better safety profile as compared to nanoparticles, different methods of synthesis were proposed to obtain magnetite microparticles with superparamagnetic properties (Ma and Chen, 2016), still each technique presents its limitations, and to the best of our knowledge, it was not described yet the method that fulfills all the requirements for biocompatible Fe_3_O_4_ microparticles. IOPs have an iron oxide core, which is covered by polyethylene glycol, dextran, or other biocompatible materials. For this reason, their size is generally referred to as the hydrodynamic diameter. Below 10 nm, the magnetization exhibits thermally activated fluctuations inside the particle core from an easy axis of magnetization to another (e.g., the <111> directions in the case of magnetite), which leads to superparamagnetic relaxation (Néel, 1949). Thus, the particles do not remain agglomerated after field removal, an important requirement in biomedical applications, not commonly found in particles larger than 10–20 nm.

In the present study it was proposed as method for Fe_3_O_4_ microparticles synthesis the hydrothermal decomposition of Fe-Na_4_EDTA complex in the presence of urea and were obtained magnetite particles with compact (not porous) single-crystalline structure, and size beyond nanometric range. The very low values of both magnetic remanence (σr=0.28 emu/g) and coercitivity (Hc=1.5 Oe), similar to nanoparticles, ensures superparamagnetic behavior. In addition, as the magnetic saturation is very close to the bulk value (σs=92 emu/g), these particles are highly magnetizable. Bean and Livingston (1959) showed that large particles (larger than 15 nm) are not superparamagnetic (Bean and Livingston, 1959), but they can easily combine as a solution to form a superparamagnetic aggregate, the magnetic saturation of the nanoparticles is diminished (commonly to 30 emu/g to 50 emu/g) due to the greater fraction of metal ions located on the crystal surface (Cornell and Schwertmann, 2003; Yuan et al., 2012). Also, spontaneous oxidation in air yields γ-Fe_2_O_3_ with additional loss (up to 20%) of magnetic saturation (Kucheryavy et al., 2013). To increase the transverse relaxivity, Berret et al. (2006) fabricated maghemite aggregates in the range of 70–150 nm by using block copolymers template (Berret et al., 2006). Shapiro et al. (2004) showed that MIOPs carry iron at an order of magnitude greater than USPIOs and can cause a local magnetic field in homogeneity extending approximately 50 times beyond the physical diameter of the particle. Accordingly, such particles could be suitable for biomedical applications (Shapiro et al., 2004).

**Figure 8:**
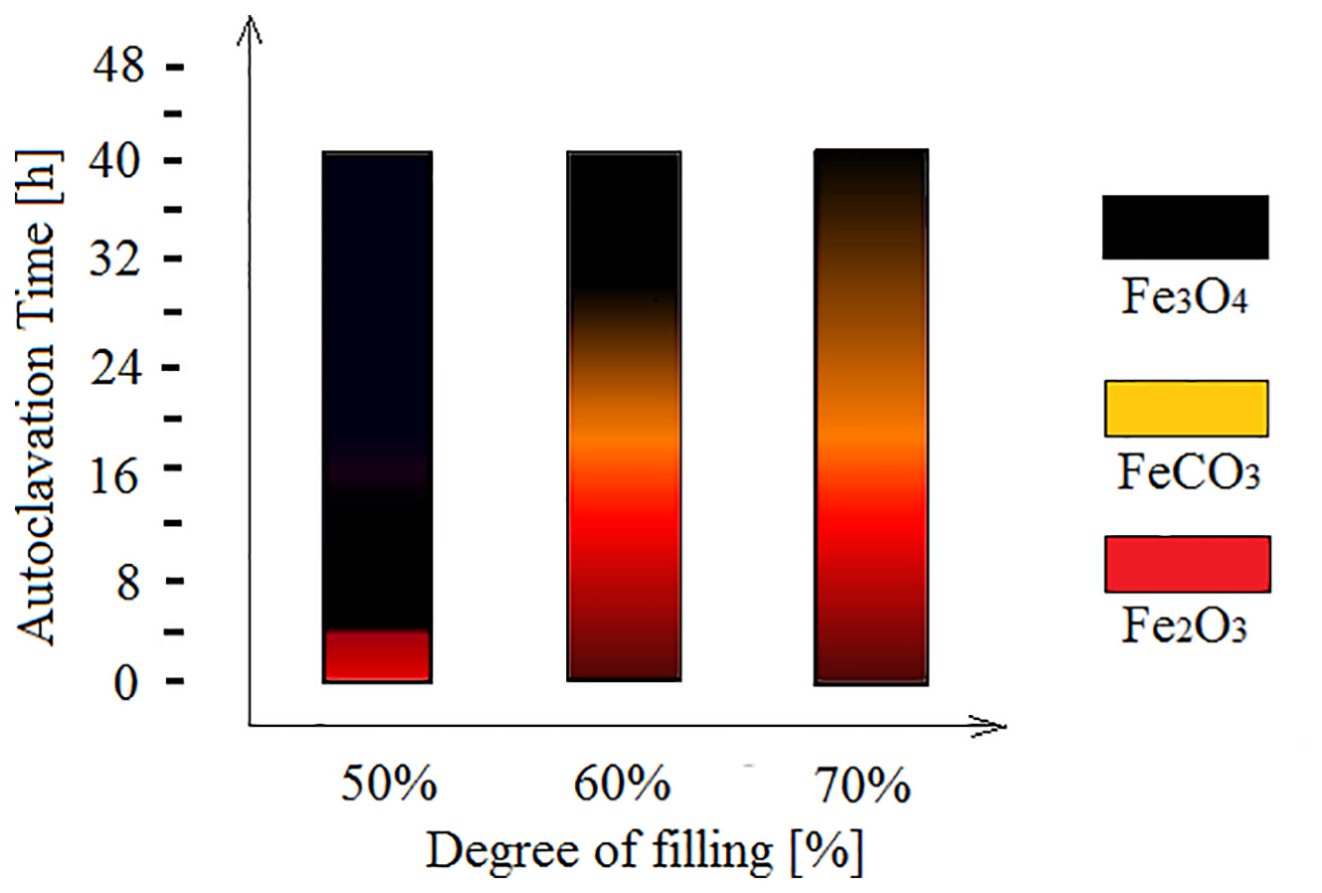
Final products depending on degree of autoclaves filling.

Regarding our synthesis, the major concern was to eliminate the secondary synthesis product, iron carbonate (FeCO_3_), that appears in the form of individual rhombohedral single-crystals alongside the magnetite. Therefore, the particle mixture was washed in a solution of hydrochloric acid (HCl) to ensure its removal. The solution concentration, the washing time and temperature were chosen not to affect the integrity of the magnetite particles. Multiple tests had shown that keeping products for three hours in a solution of HCl, pH=0.5 at 70°C leads to complete dissolution of FeCO_3_ without altering the surface of the Fe_3_O_4_ crystallites. Attempts to wash the mixture with other chemical solvents did not led to the desired results. It was noticed that the filling level of autoclaves significantly influences the synthesis processes well as the FeCO_3_ content (**Figure 8**). For a filling at 70%, the single-phase Fe_3_O_4_ with an average size of 40 µm was obtained after about 40 h of high-pressure treatment time. Otherwise, only mixture of α-Fe_2_O_3_, Fe_3_O_4_, and FeCO_3_ was obtained. For a filling at 60%, the same mixture of α-Fe_2_O_3_, Fe_3_O_4_, and FeCO_3_ was obtained except at 32, 34, 36, 38, and 40 h of high-pressure treatment time. Single-phase magnetite was obtained around 30 µm, at such instances. When the filling level was reduced to 50% or less, only a single phase of magnetite was obtained with no trace of FeCO_3_ for any 4 to 40 h treatment times. Full control of the particle size growing progressively from 1 µm to 30 µm is so achieved and no HCl-based washing is needed.

In order to verify the uncoated SCMIOPs’ potential applications to biomedical field *in vitro* cytocompatibility/cytotoxicity tests were performed on tumor and non-tumor cell lines. Iron oxide particles (particularly of nanoscale size) can promote cellular damage in human and mammalian cells characterized by DNA damage, disruption of cytoskeleton, apoptosis and oxidative stress by inducing generation of ROS (reactive oxygen species) *via* Fenton reaction (Dissanayake et al., 2015). The results indicate that the 24 h direct contact of SCMIOPs with HaCaT cells led to a decrease of cell viability in a dose-dependent manner. The lowest viability rate (70.62%) was recorded at the highest concentration (1000 µg/ml), whereas in the case of HEMa (human primary melanocytes) cells stimulation at 250, 500, and 1000 µg/ml rendered linear viability variation above 80% viable cells (**Figure 2**). According to the ISO standard 10993-5:2009 on Biological Evaluation of Medical Devices, a compound is considered cytotoxic if reduces cell viability by more than 30% (https://www.iso.org/obp/ui/#iso:std:iso:10993:-5:ed-3:v1:en). In this context, it can be stated that the tested 1-30 µm size uncoated SCMIOPs are cytocompatible. The different behavior of HaCaT and HEMa cells to SCMIOPs’ can be attributed to: cell type (immortalized – HaCaT *vs.* primary cells – HEMa), and growth rate (HaCaT becomes 80–90% confluent within 48 h, whereas HEMa cells require more than 72 h). Moreover, it could be assumed that melanin produced by melanocytes is responsible for the constant viability rate (around 80%) of HEMa cells even at the highest concentration of SCMIOPs, since it is known that in normal conditions, melanin acts as an iron scavenger/chelator by forming complexes with iron and suppresses the iron ions potential toxicity (Ben-ShaChar and Youdim, 1992). In the case of tumor cells (particularly for murine melanoma cells - B164A5), SCMIOPs exerted a dose-dependent cytotoxic effect, with a viability rate of 49.87% calculated at the highest concentration tested – 1000 µg/ml (**Figure 3**). The response of human melanoma cells - A375 (amelanotic cells) to SCMIOPs’ impact was somehow different as compared to B164A5 cells. The viability rate at 1000 µg/ml was of 71.26%, a value that can be considered non-toxic according to the ISO standard 10993-5:2009 on Biological Evaluation of Medical Devices (https://www.iso.org/obp/ui/#iso:std:iso:10993:-5:ed-3:v1:en).

These disparities between the response of human and murine cells to stimulation may be explained by the following: (i) different origin (A375 – human *vs.* B164A5 - murine), (ii) cells phenotype (A375 – epithelial *vs.* B164A5 - mesenchymal), and (iii) metastatic potential (A375 – low *vs.* B164A5 - high). In addition, it could be hypothesized that melanin plays a key role in SCMIOPs induced cytotoxicity (B164A5 cells are melanin-producing cells, whereas A375 cells are amelanotic). However, the validity of this hypothesis must be further investigated. The hypothesis originates from the fact that melanin can act as a double-edge sword in brain tissue. It can act as a protector by scavenging iron ions (in normal conditions) and as a promoter of Fe^3+^induced toxicity (synergistic effect by increasing the production of ROS), which can lead to neurodegenerative damage (Zucca et al., 2017). Further studies are required to verify if the iron-melanin interactions induce similar effects in melanoma cells as the ones described for brain cells.

One of the mechanisms described for iron oxides cytotoxicity was induction of apoptosis with cytoskeleton reorganization and nuclear fragmentation (Dissanayake et al., 2015; Moaca et al., 2018). DAPI staining assay was performed in order to identify the type of cytotoxicity exerted by SCMIOPs. The presence of nuclear fragmentation observed in all cell types (mainly in tumor cells - **Figures 4** and **5**) after stimulation with SCMIOPs at 150 µg/ml confirms that the uncoated particles induced apoptosis in a dose-dependent manner. Therefore, the results confirm cell viability data. Cell morphology changes were also observed after stimulation with concentrations larger than 100 µg/ml (bright field images - **Figures 4** and **5**). A very relevant feature of iron oxide particles in terms of biocompatibility and effectiveness, is represented by their capacity to be internalized by cells (Catalano, 2017). However, the cellular uptake of iron oxide particles depends on various factors such as: cell type, surface charge, cell size and coating agents (Zhou et al., 2017). In this respect, the intracellular accumulation of uncoated SCMIOPs was assessed by Prussian blue staining method. As shown in **Figures 6** and **7**, the internalization of the uncoated SCMIOPs was rather absent in the case of HaCaT and A375 cells, whereas in the case of HEMa and B164A5 cell lines, the cell membrane adhesion is more likely as opposed to particle cell internalization. The cell iron penetration mechanism described in the literature is clathrin-mediated endocytosis *via* transferrin carrier. According to several experimental studies, 50 nm is the optimum nanoparticle size needed to reach the highest cellular uptake. However, this value is also cell-type dependent (Hoshyar et al., 2016; Behzadi et al., 2017). Other studies showed that 500 nm particles can be internalized by cells *via* clathrin-mediated endocytosis (Chaves et al., 2017). Hinds et al. (2003) showed that MIOPs can be effectively endocytosed by various cells and can thus contribute to the improvement of MRI (magnetic resonance imaging) signal (Hinds et al., 2003). By coating this particles with a polymer and further label them with a fluorescent agent, they can be used both for the improvement of MRI imaging and for tracking cells by fluorescence microscopy. In a recent study, Chaves et al. (2017) showed that maghemite nanoparticles were more intensively internalized by breast cancer cells as compared to normal cells (Chaves et al., 2017). In addition, the invasive breast metastatic cells - MDA-MB-231 presented a higher uptake of the nanoparticles as MCF7 cells (non-metastatic cells). These results are similar with our data. A possible explanation for the lack of intracellular SCMIOPs accumulation could be the reduced 24 h incubation time. Jarockyte et al. (2016) noticed that for the cellular uptake of superparamagnetic magnetite nanoparticles a 24–48 h incubation time was required, during the first hours the nanoparticles were attached to cell membranes (Jarockyte et al., 2016), a phenomenon that was observed in HEMa and B164A5 cells. The coating agent on the particle surface may facilitate particle access within the cells. Hence, the lack of the agent in the uncoated particles may be responsible to some degree to the absence of SCMIOPs intracellular accumulation noted in our study. Similar results were also described in a recent study published by our research group which revealed that naked Fe_3_O_4_ nanoparticles did not affect the viability of HaCaT, B164A5 and A375 cells, up to a concentration of 50 µg/ml, whereas the presence of oleic acid (OA) coating on the surface of Fe_3_O_4_ nanoparticles led to damage of melanoma cell lines (B164A5 and A375 cells) at a concentration of 10 µg/ml, whereas the viability of HaCaT cells was affected only at the highest concentration - 50 µg/ml (Moaca et al., 2019). Comparable data were reported by Marcus et al. (2016) who evaluated the effect of different MNPs coatings on neuronal cells (Marcus et al., 2016).

## Conclusions

Developing a synthesis pathway for obtaining 1–30 µm magnetite micro octahedrons with superparamagnetic behavior (Hc=1.5 Oe, σr=0.28 emu/g) at room temperature by hydrothermal decomposition of Fe-Na_4_EDTA complex in the presence of urea led to a saturation magnetization like in bulk (σs=92 emu/g), that guarantees a strong magnetic response. Such behavior is very unusual in the case of monocrystalline particles with micrometric dimensions which should have a typical ferrimagnetic rather than superparamagnetic behavior.

Present *in vitro* findings reveal that the uncoated magnetite microparticles are cytocompatible/cytotoxic in a cell-dependent manner on non-tumor/tumor cells accordingly, in agreement to ISO standard 10993-5:2009. In the case of HaCaT cells (non-tumor cells with a high proliferation rate) no microparticle accumulation within the cells was observed, however a dose-dependent decrease of cells viability resulted. For HEMa cells (low proliferation rate melanin-producing non-tumor cells) microparticles adhered to the cell membrane and the effect on cell viability becomes linear at concentrations higher than 150 µg/ml (above 80%). In case of B164A5 cells (melanin-producing murine metastatic melanoma cells), microparticles adhered to cells membranes and induced cytotoxicity *via* apoptosis in a dose-dependent manner. Regarding A375 cells (amelanotic non-metastatic human melanoma cells), no intracellular accumulation of the microparticles was detected and the cytotoxic effect was less pronounced when compared to B164A5 cells. The results indicate that SCMIOPs are cytocompatible for non-tumor cells with a low proliferation rate and cytotoxic for tumor metastatic cells, hypothesizing that melanin is the key player in the mechanism of action involved. Here was demonstrated that a new class of micrometric magnetite particles overcome the limitations in using magnetic nanoparticles and micro-clusters. These new reported SCMIOPs possess a difference between magnetic saturation and magnetic remanence of 95.5 emu/g. Carefully selecting their size, it could be possible that applications like cellular MRI, monitoring cell migration for cell therapy, MRI contrast agents, detection, immobilization, and modification of biologically active compounds, cell labeling, magnetic separation of cells and others, to benefit from using SCMIOPs.

## Data Availability Statement

The datasets generated for this study are available on request to the corresponding authors.

## Author Contributions

CF, IM, IP, and DC – conception of the study, performed the *in vitro* tests, analysis and interpretation of the data acquired, drafting the work, and prepared the manuscript for submission. MCC, MC, LM, and AI – performed the synthesis, analysis and interpretation of the data acquired related to iron microparticles, and drafting the work. SA – performed *in vitro* tests, analysis of the results, and drafting the work. IP, CD, FL, VR, and AE – elaboration of the final version of the manuscript, correction of the language, analysis of the data, and revised critically the work. All authors contributed to manuscript revision, read, and approved the submitted version.

## Funding

This work was supported by a grant of Ministery of Research and Innovation, CNCS-UEFISCDI, project number PN-III-P1-1.1-PD-2016-1982, within PNCDI III - lines 1109-1111 and by a grant offered by “Iuliu Hatieganu” University of Medicine and Pharmacy, Cluj-Napoca PCD contract no. 3066/17/01.02.2018, awarded by Claudia Geanina Farcaș.

## Conflict of Interest

The authors declare that the research was conducted in the absence of any commercial or financial relationships that could be construed as a potential conflict of interest.
